# A conserved motif is essential for the correct assembly of proglutelins and for their export from the endoplasmic reticulum in rice endosperm

**DOI:** 10.1093/jxb/ery290

**Published:** 2018-08-10

**Authors:** Lihong Tian, Yanping Xing, Masako Fukuda, Rong Li, Toshihiro Kumamaru, Dandan Qian, Xiangbai Dong, Le Qing Qu

**Affiliations:** 1 Key Laboratory of Plant Molecular Physiology, Institute of Botany, the Chinese Academy of Sciences, Beijing, China; 2 Faculty of Agriculture, Kyushu University, Fukuoka, Japan

**Keywords:** Disulfide bonds, endoplasmic reticulum export, glutelin, rice endosperm, sequence motif, protein assembly

## Abstract

Rice glutelins are initially synthesized as 57-kDa precursors at the endoplasmic reticulum (ER) and are ultimately transported into protein storage vacuoles. However, the sequence motifs that affect proglutelin folding, assembly, and their export from the ER remain poorly defined. In this study, we characterized a mutant with nine amino acids deleted in the GluA2 protein, which resulted in specific accumulation of the GluA precursor. The deleted amino acids constitute a well-conserved sequence (LVYIIQGRG) in glutelins and all residues in this motif are necessary for ER export of GluA2. Immunoelectron microscopy and stable transgenic analyses indicated that proglutelins with deletion of this motif misassembled and aggregated through non-native intermolecular disulfide bonds, and were deposited in ER-derived protein bodies (PB-Is), resulting in conversion of PB-Is into a new type of PB. These results indicate that the conserved motif is essential for proper assembly of proglutelin. The correct assembly of proglutelins is critical for their segregation from prolamins in the ER lumen, which is essential for enabling the export of proglutelin from the ER and for the proper formation of PB-Is. We also found that the interchain disulfide bond between acidic and basic subunits is not necessary for their assembly, but it is required for proglutelin folding.

## Teaser

Characterization of a mutant that specifically accumulates the GluA precursor reveals a conserved motif in proglutelins that is essential for their proper assembly in, and their subsequent export from, the endoplasmic reticulum.

## Introduction

Endosperm storage proteins in rice (*Oryza sativa*) comprise mainly alcohol-soluble prolamins, acid- and alkaline-soluble glutelins, and saline-soluble α-globulin. Prolamins are synthesized on the ER and form spherical protein bodies, PB-Is (Bechtel and Juliano, 1980; Li *et al.*, 1993). Glutelins, accounting for 60–80% of the total protein, are encoded by a multigene family and are classified into four subfamilies: GluA, -B, -C, and -D. The GluA and GluB subfamilies are composed of at least three and five members, respectively (Takaiwa *et al.*, 1991; Kusaba *et al.*, 2003; Kawakatsu *et al.*, 2008). As a member of the 11S globulin family, glutelins are synthesized as 57-kDa proglutelins (also called glutelin precursors) at the rough ER (rER) and are then transported to the protein storage vacuoles (PSVs, also called protein body IIs, PB-IIs) through the Golgi apparatus and dense vesicles (DVs). The proglutelins are ultimately cleaved into ~40-kDa acidic (α) and ~20-kDa basic (β) subunits and deposited together with α-globulin in the PSVs (Krishnan *et al.*, 1986; Yamagata and Tanaka, 1986). Any defects in the glutelin folding, sorting, and transport processes will lead to high-level accumulation of the 57-kDa proglutelin, referred to as the 57H phenotype. Thus, 57H mutants provide ideal material for investigating the machinery of trafficking of storage proteins.

Many studies of glutelin intracellular transport in 57H mutants have identified regulatory factors related to movement from the Golgi apparatus to the PSVs and processing within the PSVs. The small GTPase Rab5a and its two guanine exchange factors, VPS9a and GEF2, participate in the transport of proglutelin from the Golgi to the PSVs and are involved in the maintenance of the general structure of endomembrane system (Wang *et al.*, 2010; Fukuda *et al.*, 2011, 2013; Liu *et al.*, 2013; Wen *et al.*, 2015). GPA3 forms a regulatory complex with Rab5a and VPS9a and controls DV-mediated post-Golgi trafficking of major storage proteins (Ren *et al.*, 2014). Vacuolar-processing enzyme 1 (VPE1) proteolytically processes proglutelins into acidic and basic subunits, which is essential for the formation of proper crystalline structures in PB-IIs (Wang *et al.*, 2009; Kumamaru *et al.*, 2010).

The ER is the first compartment of the secretory pathway and is the site where protein synthesis, folding, and assembly take place (Mikosch and Homann, 2009). After synthesis on the rER, correct folding and assembly of proteins are crucial for their sorting (Liu and Li, 2014). Disulfide-bond formation is necessary for folding and assembly of many storage proteins (Kawagoe *et al.*, 2005; Onda *et al.*, 2011). Prolamins are assembled in the ER by intermolecular disulfide bonds, which are essential for stabilizing the PB-I structure. The proglutelins are folded through intramolecular disulfide bonds before being transported from the ER. Knockdown of the oxidoreductase Ero1 inhibits the formation of native disulfide bonds, resulting in aggregation of proglutelins through non-native intermolecular disulfide bonds in the rER (Onda *et al.*, 2009). Protein disulfide isomerase (PDI) plays an essential role in the oxidative folding of vacuole-targeted storage proteins. In PDI1-1 loss-of-function mutants (e.g. *esp2*) proglutelins interact heterotypically with prolamins within the ER lumen, which leads to the formation of abnormal ER-derived PBs containing both proglutelins and prolamins (Takemoto *et al.*, 2002; Satoh-Cruz *et al.*, 2010). Correctly folded and assembled proglutelins are transported from the ER to the Golgi apparatus via coat-protein complex-II (COPII) vesicles. Disruption of COPII complex formation by simultaneous knockdown of *Sar1a*, -*b*, and -*c* expression results in the accumulation of proglutelins in the ER, yielding novel ER-derived PBs containing both glutelins and α-globulin (Tian *et al.*, 2013). GOT1B mediates the export of proglutelins and α-globulin from the ER to the Golgi via COPII vesicles (Fukuda *et al.*, 2016; Wang *et al.*, 2016).

In addition to external factors, intrinsic sequence motifs play important roles in determining the correct folding, assembly, and export of proteins from the ER. Phaseolin is a major 7S-class storage protein of the common bean and the C-terminal domain and internal region are necessary for trimeric assembly (Pedrazzini *et al.*, 1994, 1997; Foresti *et al.*, 2003). However, our understanding of the sequence motifs in rice proglutelins that determine their folding, assembly, and export from the ER remains limited. In this study, we identified a well-conserved sequence involved in the correct assembly of proglutelins through characterization of a 57H mutant and transgenic lines. Our results indicated that a conserved nine-residue motif, LVYIIQGRG, is essential for proper assembly of proglutelin in the ER lumen and for subsequent export from the ER. We also found that the interchain disulfide bond between acidic and basic subunits is not necessary for proglutelin assembly but is required for its folding.

## Materials and methods

### Plant material and growth conditions

The rice (*Oryza sativa*) EM1317 mutant line was induced by *N*-methyl-*N*-nitrosourea (MNU) mutagenesis as described previously (Kumamaru *et al.*, 1988). The wild-type was Taichung 65 (TC65). All plants were grown in fields during normal growing seasons or in a greenhouse at the Institute of Botany, Chinese Academy of Sciences, Beijing, China.

### Protein extraction, SDS-PAGE, and immunoblot analysis

The extraction of total seed proteins, SDS-PAGE, and immunoblot analysis were performed as described by Tian *et al.* (2013). Isoelectric focusing (IEF) of glutelin was performed as described previously (Qu *et al.*, 2002). Sequential extraction of proteins was performed according to Takemoto *et al.* (2002).

To detect disulfide-bond formation, proteins were extracted from mature seeds as described previously by Onda *et al.* (2009). For protein fractionation, proteins were extracted from mature rice seeds in non-reducing buffer B (10% glycerol, 4% SDS, 8 M urea, and 50 mM Tris-HCl, pH 6.8) by vigorous shaking for 3 h. The homogenate was centrifuged, and the supernatants were collected (fraction S). The resulting pellets (fraction NR-P) were homogenized in buffer B containing 100 mM DTT for 30 min, and the soluble fractions were collected by centrifugation (fraction P). Total proteins (fraction T) were extracted from mature seeds in buffer B containing 100 mM DTT. The S fractions were reduced with 100 mM DTT before SDS-PAGE analysis. The NR-P fractions were homogenized in buffer B with varying concentrations of DTT (0, 1, 3, and 10 mM). After centrifugation, the supernatants were subjected to 5–20% gradient SDS-PAGE.

Sucrose density gradient (SDG) centrifugation was performed as described by Wakasa *et al.* (2009) with modifications. A sample of 1 g dehulled developing rice seeds was ground with 10 mM Tris–HCl, pH 6.8, containing 0.5 M NaCl. The homogenate was filtered through cheesecloth and centrifuged at 100 *g* for 10 min. The supernatant was layered onto linear 10–30% SDGs on a 65% sucrose cushion. After centrifugation at 120 000 *g* for 16 h, fractions (500 μl each) were successively collected from the bottom and precipitated with acetone. The precipitate was used for SDS-PAGE and immunoblot analyses.

### Determination of grain amino acid contents

To determine the amino acid content and composition, extracts were prepared from 100 mg of finely ground seeds and examined using HPLC according to Sanders *et al.* (2009).

### Microscopy observations

Transmission electron microscopy and immunogold localization were performed as described previously (Kumamaru *et al.*, 2010; Tian *et al.*, 2013).

### Gene cloning and sequencing

Rice genomic DNA was extracted from leaves using the cetyl trimethyl ammonium bromide method. The coding regions of *GluA1*, *GluA2*, and *GluA3* together with the promoter and 3′-untanslated region (3′-UTR) were amplified by PCR. Total RNA was isolated from developing seeds using the TRIpure reagent method (Bioteke, China), and first-strand cDNA was generated as a template for amplification of *GluA2* coding sequences. The PCR products were cloned into the pMD18-T vector (Takara Bio Inc., Japan), and inserts were sequenced.

### Vector construction and rice transformation

To construct fusion proteins for rice transformation, the *GluA2/mGluA2-3FLAG* fragments were inserted into the binary vector pGPTV under the control of the endosperm-specific *GluA2* promoter. The deletion-mutant fragments were amplified from *GluA2-3FLAG* (cloned into pMD18-T) using a Takara MutanBEST kit and then inserted into pGPTV. *GluA1*, *GluB4*, and corresponding deletion mutants were prepared using the same method. The constructs were introduced into rice (*O. sativa* cv. Kitaake) by *Agrobacterium tumefaciens*-mediated transformation. Successful transformants were verified by PCR as described previously (Qu *et al.*, 2008).

To determine subcellular localization in rice protoplasts, green fluorescent protein (GFP) was fused to the C-terminus of *GluA2* and the deletion mutants. The chimeric genes were subcloned into pBI221 under the control of the CaMV 35S promoter to obtain transient expression vectors, which were co-transformed into protoplasts. Transformed cells were examined under a confocal microscope, and digital images were recorded. Three-dimensional reconstruction functions were employed as described by Tian *et al.* (2013). More than 30 protoplast cells were observed for each construct study.

All primer sequences used for PCR are listed in Supplementary Table S1 at *JXB* online.

## Results

### GluA subfamily proglutelin accumulates specifically in the EM1317 mutant line

**Fig. 1.**
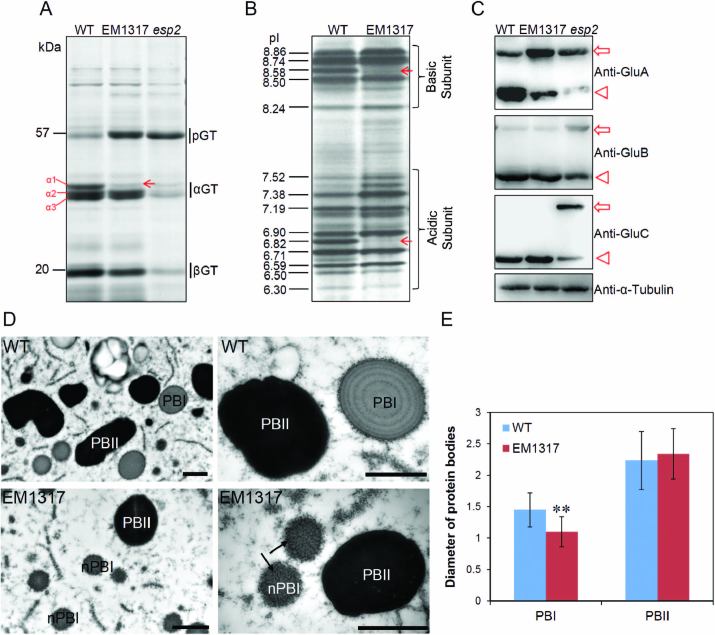
Phenotypic analyses of the EM1317 mutant. (A) SDS-PAGE analysis of storage proteins in mature seeds from the wild-type, and EM1317 and *esp2* mutants. pGT, glutelin precursors; αGT, glutelin acidic subunits; βGT, glutelin basic subunits. The arrow indicates the decreased glutelin α-1 subunit. (B) Isoelectric focusing analysis of glutelin composition from the wild-type and EM1317 mutant. Arrows indicate the bands that disappeared in EM1317. (C) Immunoblot analysis of the glutelin subfamily proteins (GluA, GluB, and GluC); α-tubulin was used as a loading control. Arrows indicate the 57-kDa proglutelins, while arrowheads indicate the glutelin acidic subunits. (D) TEM of protein bodies in the wild-type and EM1317 mutant. Arrows indicate the new type of PB-I (nPB-I). Scale bars are 1 μm. (E) Diameters of PB-I and PB-II. Data are means (±SD), *n*>100. **P<0.01 (Student’s *t*-test).

The mutant line EM1317 was obtained by screening progeny derived from the treatment of fertilized egg cells of rice (*O. sativa* cv Taichung 65) with MNU. The seeds exhibited no visible abnormalities in morphology (Supplementary Fig. S1A). Compared with the wild-type, the level of 57-kDa proglutelins was markedly increased and the 40-kDa α-1 subunit was markedly decreased in the EM1317 line, while the levels of the α-2 and α-3 subunits were not altered (Fig. 1A). IEF analysis of glutelin fractions indicated that an acidic pI6.82 band and a basic pI8.58 band disappeared in EM1317 (Fig. 1B). Immunoblot analysis with glutelin isoform-specific antibodies indicated accumulation of the GluA proglutelins, accompanied by a decrease in the acidic subunits in the EM1317 mutant. The proglutelins and mature acidic subunits of the GluB and GluC subfamilies showed no differences between EM1317 and the wild-type (Fig. 1C). The expression levels of prolamins and α-globulin were not affected (Supplementary Fig. S1B) and the concentrations of 15 types of amino acid in the seeds did not change (Supplementary Table S2).

### A new type of ER-derived PB is formed in the EM1317 mutant line

To determine whether the accumulation of proglutelin in EM1317 affected PB formation, we examined the intracellular structures of developing endosperm cells by TEM. In the wild-type, ER-derived prolamin-containing PB-Is were spherical, electron-lucent, and surrounded by rough ER membrane with ribosomes, showing concentric rings of varying electron density, whereas glutelin- and α-globulin-containing PB-IIs were irregularly shaped with high electron density, exhibiting uniform staining (Fig. 1D). Interestingly, in the EM1317 line, we observed normal appearance of PB-IIs but did not find the typical PB-Is; instead, a new type of PB was evident. Similar to PB-Is, these PBs were spherical with polysomes attached to the surface but were much smaller and lacked the concentric ring structure. The attachment of the polysomes suggested that the new type of PB was derived from the same ER that gave rise to PB-Is. We named the new type of PB as nPB-I (Fig. 1D). The mean size of nPB-Is was significantly smaller than that of PB-Is in the wild-type, whereas there were no obvious differences in the size of PB-IIs (Fig. 1E).

### Some glutelins are distributed in nPB-Is

**Fig. 2.**
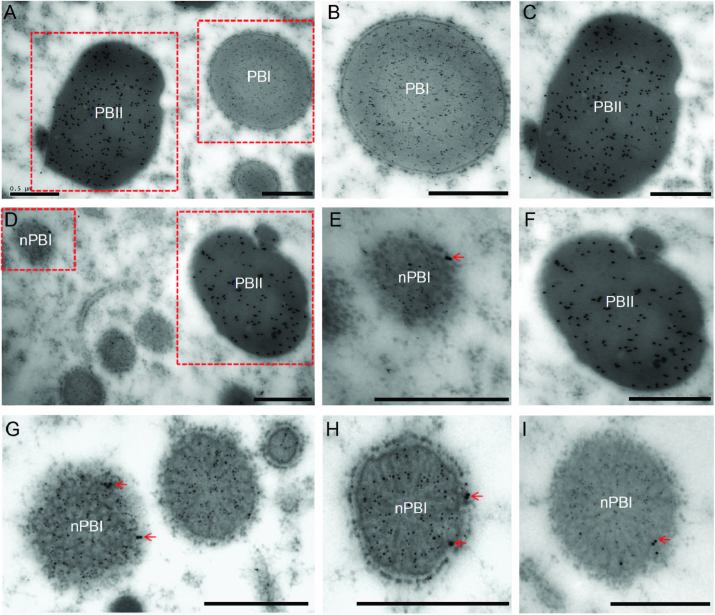
Immunolocalization of glutelin and prolamin in protein bodies. (A–C) Wild-type and (D–I) EM1317 mutant. (B, C, E, F) show enlarged images of the boxed areas in (A, D). Glutelin and prolamin antibodies were labelled with 15- and 5-nm immunogold particles, respectively. Arrows indicate glutelins accumulated in nPB-Is. Scale bars are 0.5 μm.

The appearance of nPB-Is in the EM1317 mutant was very similar to that of the ER-derived inclusion body PBs in the *esp2/pdi1-1* mutant (Takemoto *et al.*, 2002); both contained normal PB-IIs but lacked normal PB-Is. Therefore, the intracellular localization of glutelins and prolamins was examined by immunoelectron microscopy (IEM). In the wild-type, glutelins labelled with 15-nm immunogold particles were distributed in PB-IIs, and prolamins labelled with 5-nm immunogold particles were detected in PB-Is (Fig. 2A–C). However, in the EM1317 mutant, in addition to being distributed in normal PB-IIs, glutelins were also observed in the nPB-Is, while prolamins were detected only in the nPB-Is (Fig. 2D–I). These observations indicated that some glutelins that were blocked in the ER were deposited with prolamins, resulting in the conversion of PB-Is to a new type of PB in the EM1317 mutant.

### The structural gene *GluA2* is defective in the EM1317 mutant line

Although the ER-derived inclusion body nPB-Is in the EM1317 and *esp2* mutants had similar morphologies, the glutelin profiles were different. In *esp2* the levels of all mature glutelin acidic and basic subunits decreased coupled with increases in multiple proglutelins (Fig. 1), while in EM1317 only the α-1 subunit decreased coupled with an increase in the GluA subfamily proglutelin level, and the amounts of other subunits did not change. These results suggested that EM1317 may be a mutant defective in a glutelin structural gene and not a regulatory gene mutation similar to *esp2*.

**Fig. 3.**
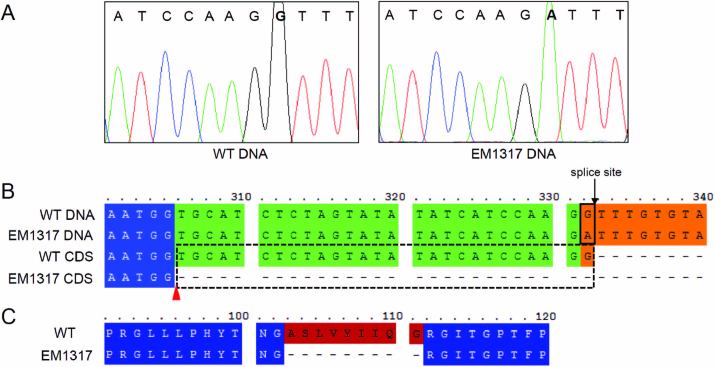
Mutation site analysis of GluA2 in the EM1317 mutant. (A) Sequence analysis reveals a single guanine (G)-to-adenine (A) nucleotide substitution in the *GluA2* DNA sequence of the EM1317 mutant. (B) Nucleic acid sequence alignment of *GluA2* indicates that the mutation site (boxed by the solid line) is an exon/intron splice site. The black arrow indicates the splice site in the wild-type, while the red arrowhead indicates the splice site in the EM1317 mutant. The dashed lines indicate the deleted nucleic acids. (C) Amino acid sequence alignment of GluA2 in the wild-type and EM1317 mutant.

Genomic DNA sequencing of *GluA* subfamily members revealed a nucleotide mutation in the *GluA2* (LOC_Os10g26060) gene of EM1317. As shown in Fig. 3, the gene sequence revealed that EM1317 contained a single-nucleotide substitution in which a guanine (G) was replaced by an adenine (A) at the 5′-splice site of the first intron. Sequence alignment showed that the splice site was skipped to a position 27 bp upstream of the constitutive splice site, which resulted in a 27-nucleotide deletion of *GluA2* cDNA in EM1317 (Fig. 3B, line 4). The deleted nucleotides led to a nine amino acid (_103_ASLVYIIQG_111_) deletion of the corresponding protein (Fig. 3C). The other members of the *GluA* subfamily and the promoter region (2.3 kb) and 3’-UTR (1.1 kb) sequences were analysed, and no other nucleotide mutations were detected in the EM1317 mutant.

### The ASLVYIIQG deletion is responsible for the mutated GluA2 blocked in the ER

To verify which step of glutelin transport was affected by deletion of the ASLVYIIQG peptide, *GluA2-GFP* and ASLVYIIQG-deleted *mGluA2-GFP* constructs were transiently expressed with the ER marker *mCherry-HDEL*, the Golgi marker *Man1-mCherry*, the trans-Golgi network (TGN) marker *mRFP-SYP61*, and the prevacuolar compartment (PVC) marker *mRFP-VSR2* (Wang *et al.*, 2014) in rice protoplasts (Supplementary Fig. S2). The results showed that GluA2-GFP was delivered to the vacuole with punctate structures in protoplasts (Supplementary Fig. S3A). The GluA2-GFP vacuolar expression pattern was similar to that of aleurain, a vacuolar cargo protein, with a diffuse fluorescence signal in the vacuole (Zeng *et al.*, 2015). The punctate signals of GluA2-GFP were partially co-localized with the TGN and PVC markers. mGluA2-GFP was fully co-localized with the ER marker mCherry-HDEL, with no punctate fluorescent signals or vacuolar distribution. Immunoblot results showed that the intact fusion proteins were expressed in the protoplast cells (Supplementary Fig. S3B). These results indicated that GluA2-GFP was delivered from the ER to vacuoles along the secretory pathway, passing through the Golgi, TGN, and PVC in protoplasts. The retention of mGluA2-GFP in the ER suggested that the deleted ASLVYIIQG was essential for the export of GluA2 from the ER.

**Fig. 4.**
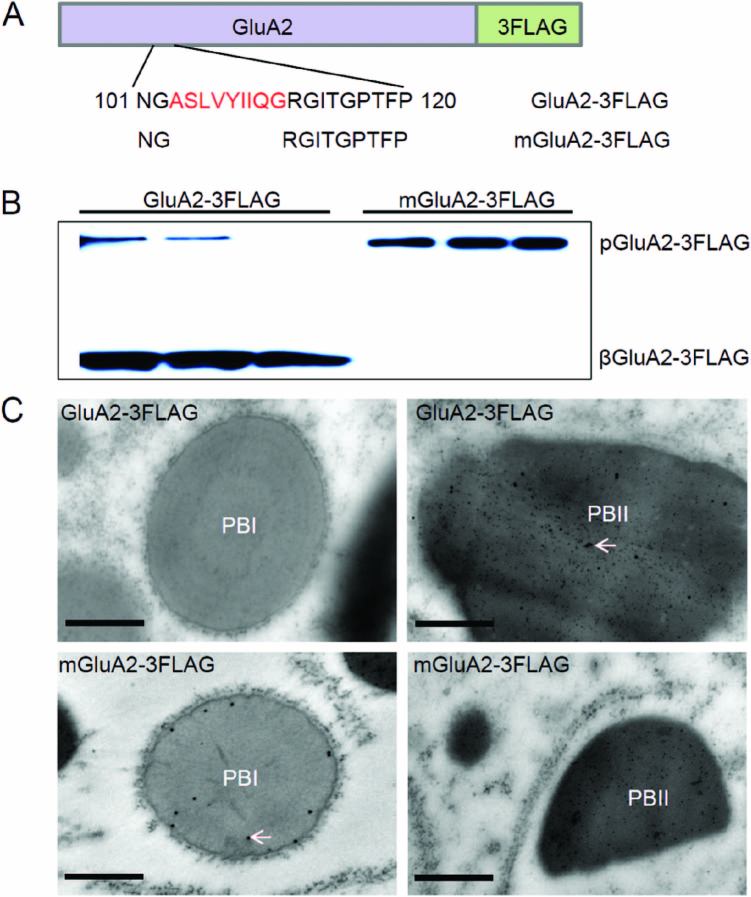
Subcellular localization of the GluA2-/mGluA2-3FLAG fusion protein in transgenic endosperm. (A) Diagram of the GluA2-/mGluA2-3FLAG constructs for transgenic rice. (B) Immunoblotting detects fusion protein expression using an anti-FLAG antibody in three different transgenic lines. pGluA2, GluA2 precursors; βGluA2, GluA2 basic subunits. (C) Immunolocalization of the fusion proteins in transgenic endosperm cells. The glutelin and FLAG antibodies were labelled with 5-nm and 15-nm immunogold particles, respectively. Arrows indicate the 15-nm immunogold particle-labelled fusion protein of two different constructs in the distinct protein bodies. Scale bars are 0.5 μm.

Although wild-type GluA2 protein was distributed in the vacuoles of rice protoplasts, we did not find PSVs in these cells. This may have been due to the difference between protoplasts and endosperm cells. Therefore, we generated transgenic rice specifically expressing GluA2-3FLAG and mGluA2-3FLAG in the endosperm under the control of the *GluA2* promoter (Fig. 4A). In *GluA2-3FLAG* transgenic lines most of the fusion proteins exhibited the β subunit fused with 3FLAG bands of ~30 kDa, while some were ~60 kDa, which corresponded to the GluA2 proglutelin fused with 3FLAG as expected. However, in m*GluA2-3FLAG* transgenic lines the expressed protein was identified as the 60 kDa GluA2 proglutelin, while the 30 kDa β-subunit could not be detected (Fig. 4B). These results indicated that the GluA2-3FLAG protein could be transported successfully into PSVs and proteolytically processed into α and β mature subunits, while the mGluA2-3FLAG remained as proglutelin.

There are two possible explanations for mGluA2-3FLAG proglutelin accumulation: the proglutelins may have been sorted into PSVs but were defective in processing by VPE1, or the proglutelins may have failed to arrive in the PSVs. To determine which was the case, we examined the subcellular localization of the transferred protein. In *GluA2-3FLAG* transgenic cells, the 15-nm immunogold particle-labelled fusion proteins accumulated mainly in irregularly shaped PB-IIs, and no signals were detected in PB-Is, similar to the expression pattern of glutelins in the wild-type. However, in m*GluA2-3FLAG* transgenic cells, the anti-FLAG immunogold particles were only detected in PB-Is, which also lacked a concentric ring structure, similar to nPB-Is in the EM1317 mutant (Fig. 4C). The subcellular localization of the fusion proteins confirmed that GluA2 without ASLVYIIQG was blocked in the ER. These results indicated that ASLVYIIQG was necessary for the export of GluA2 from the ER.

### The deleted amino acids are conserved in the glutelin family

**Fig. 5.**
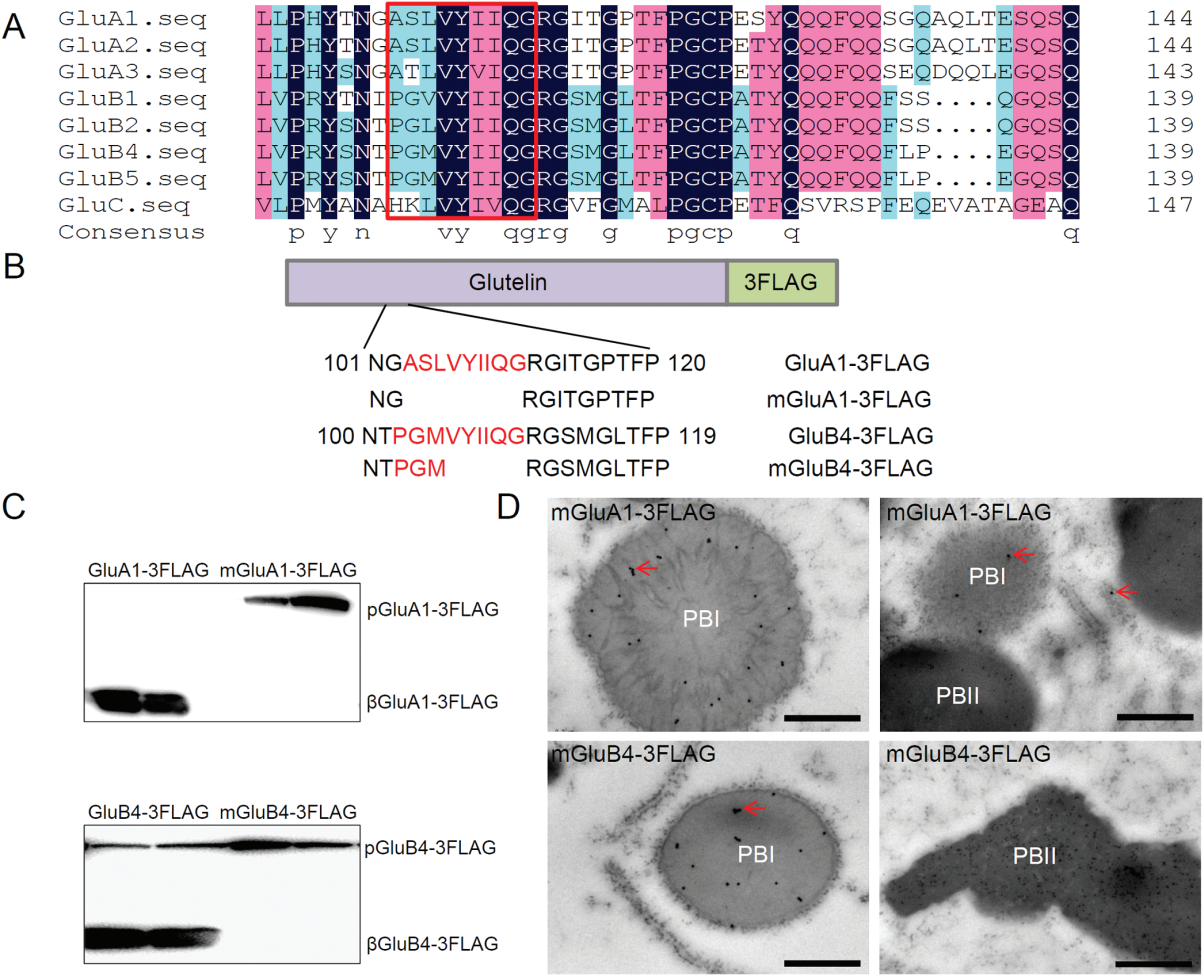
The nine deleted amino acids are conserved in glutelin family members. (A) Amino acid sequence alignment of the glutelin family. Boxed letters indicate the nine deleted amino acids. (B) Diagram of GluA1, GluB4, and mutated protein constructs for transgenic rice. (C) Immunoblotting detects fusion protein expression with anti-FLAG antibody in two different transgenic lines. pGluA1, GluA1 precursors; βGluA1, GluA1 basic subunits; pGluB4, GluB4 precursors; βGluB4, GluB4 basic subunits. (D) Immunolocalization of the fusion proteins in transgenic endosperm cells. The glutelin and FLAG antibodies were labelled with 5-nm and 15-nm immunogold particles, respectively. The arrows indicate the fusion protein signals. Scale bars are 0.5 μm.

Rice glutelins are encoded by a multigene family with high sequence similarity among the members. The amino acid sequence alignment indicated that _103_ASLVYIIQG_111_ was conserved in the GluA subfamily and that _106_VYIIQG_111_ was well conserved among the GluA, GluB, and GluC subfamilies (Fig. 5A). Furthermore, two adjacent residues, Arg-112 and Gly-113, were also conserved among the glutelin subfamilies. These conserved amino acids probably play similar roles among glutelin members. To confirm this, we generated transgenic plants expressing wild-type and conserved amino acid-deleted GluA1 and GluB4 fusion proteins (Fig. 5B). The immunoblots showed that in the wild-type GluA1 and GluB4 lines the β subunits were observed, indicating that GluA1 and GluB4 could be transported to the PSVs and processed successfully. However, in transgenic seeds expressing either mGluA1 (ASLVYIIQG deleted) or mGluB4 (VYIIQG deleted), only the 60-kDa proglutelins could be detected, and the β subunits were not detected (Fig. 5C). Immunolocalization studies indicated that GluA1-3FLAG and GluB4-3FLAG proteins were distributed in PB-IIs with endogenous glutelins (Supplementary Fig. S4), while mGluA1-3FLAG and mGluB4-3FLAG proteins were accumulated mainly in PB-Is, which were similar to the nPB-Is lacking a concentric ring structure in the EM1317 mutant (Fig. 5D). These results indicated that the conserved VYIIQG motif was essential for export of the glutelin family from the ER.

### All amino acids in the LVYIIQGRG motif are necessary for export of GluA2 from the ER

To examine which amino acids in this motif were necessary for export of GluA2 from the ER, we expressed ΔASL-GFP or ΔVYIIQG-GFP in rice protoplasts (Supplementary Fig. S2). The green fluorescence of ΔASL-GFP and ΔVYIIQG-GFP merged with the red fluorescence of the ER marker mCherry–HDEL, and no punctate structures or distribution in vacuoles were observed, indicating that ΔASL-GFP and ΔVYIIQG-GFP were blocked in the ER (Supplementary Fig. S5).

**Fig. 6.**
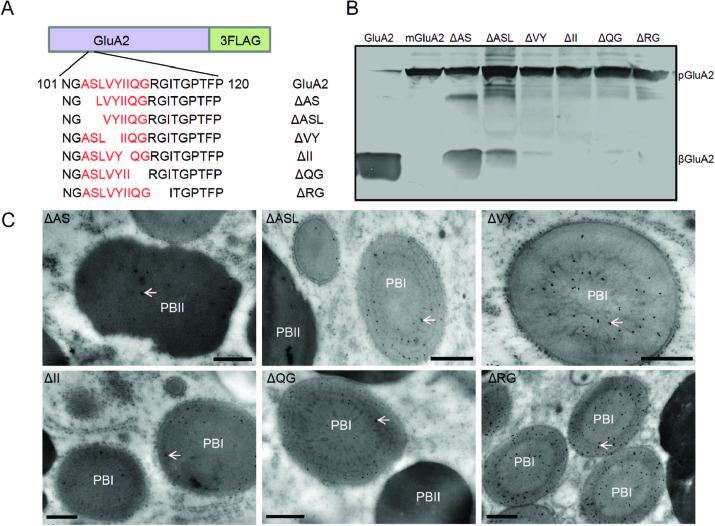
Subcellular localization of the fusion proteins with deleted amino acids in the transgenic endosperm. (A) Diagram of deleted amino acid constructs for transgenic rice. (B) Immunoblotting detects the expression with anti-FLAG antibody in different transgenic lines. pGluA2, GluA2 precursors with 3FLAG; βGluA2, GluA2 basic subunits with 3FLAG. Δ indicates the deleted amino acids. (C) Immunolocalization of the fusion protein in transgenic endosperm cells. Anti-FLAG antibody was labelled with 10-nm immunogold particles. The arrows indicate the fusion protein signals. Scale bars are 0.5 μm.

We next made a series of deletion constructs of GluA2-3FLAG to generate transgenic rice (Fig. 6A). Although the Arg-112 and Gly-113 residues were not included in the deleted amino acids ASLVYIIQG, they were conserved in glutelin subfamilies. Therefore, an RG deletion mutant was generated. Both the 30-kDa β subunit and the 60-kDa proglutelin bands were observed in ΔAS transgenic seeds, similar to GluA2-3FLAG (Fig. 6B). In contrast, ΔVY, ΔII, ΔQG, and ΔRG could not be processed into mature subunits and mainly showed 60-kDa precursor bands. Most ΔASL mutants exhibited proglutelin bands, with a small portion of the protein processed into the mature β subunit. Immunolocalization analysis revealed that the fusion protein accumulated mainly in PB-IIs with high electron density in ΔAS transgenic endosperm cells. However, in ΔASL, ΔVY, ΔII, ΔQG, and ΔRG endosperm, the mutated GluA2 proteins were mainly distributed in PB-Is with low electron density, similar to mGluA2-3FLAG (Fig. 6C). The subcellular localization of the mutated GluA2 proteins was consistent with that indicated by immunoblotting. These results demonstrated that all the amino acids in LVYIIQGRG were necessary for fusion proteins to exit from the ER, with deletion of any two amino acids resulting in the accumulation of GluA2 in the ER, but the AS residues were not necessary.

To determine whether every residue was essential for glutelin export from the ER, single-amino acid-deletion mutant constructs were expressed in rice protoplasts (Supplementary Fig. S2). As shown in Supplementary Fig. S6, Δ103A-GFP and Δ104S-GFP still reached the vacuole normally and localized to punctate structures, whereas the fluorescence of the fused proteins Δ105L-GFP, Δ106V-GFP, Δ107Y-GFP, Δ108/109I-GFP, Δ110Q-GFP, and Δ111G-GFP co-localized with that of the ER marker, mCherry-HDEL. Although Gly-102 is not located within the deleted sequence, it is close to the sequence and is conserved in the GluA subfamily. Therefore, the Gly-102 deletion construct (Δ102G-GFP) was also expressed. The results showed that Δ102G-GFP was also distributed in the vacuole, similar to GluA2-GFP (Supplementary Fig. S6). These results indicated that each of the amino acids in the LVYIIQGRG motif was essential for export of GluA2 from the ER.

### mGluA2 misassembles and forms aggregates through non-native intermolecular disulfide bonds

**Fig. 7.**
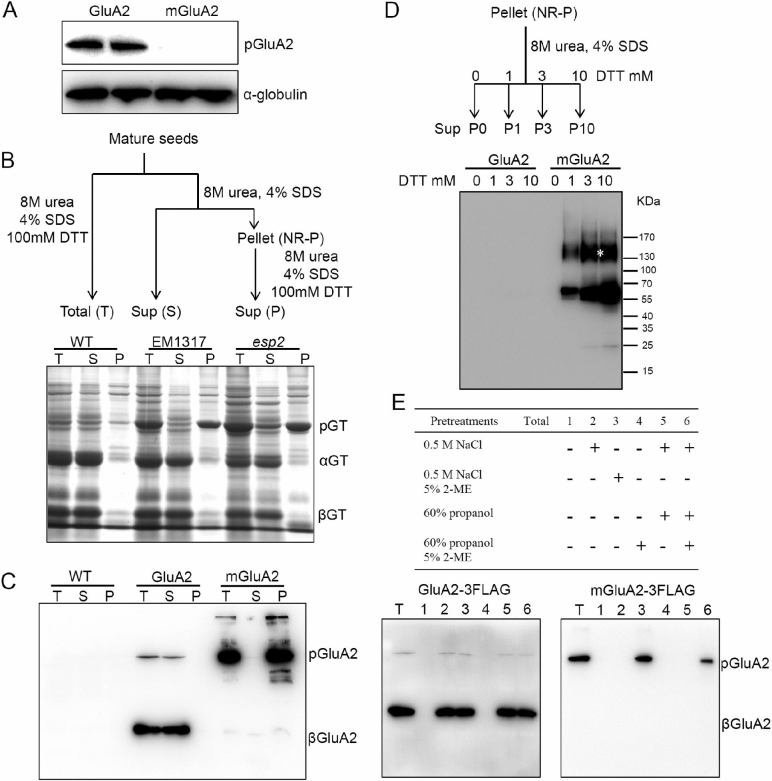
mGluA2 forms aggregates through non-native intermolecular disulfide bonds. (A) Immunoblot analysis of GluA2/mGluA2 precursors extracted by globulin extraction buffer with anti-FLAG antibody; α-globulin was used as a loading control. (B) Proteins were extracted from the mature seeds as described in the diagram (see Methods); sup, supernatant. The T, S, and P fractions were subjected to SDS-PAGE, followed by CBB staining. pGT, glutelin precursors; αGT, glutelin acidic subunits; βGT, glutelin basic subunits. (C) Immunoblotting detects the proteins of the GluA2-/mGluA2-3FLAG transgenic seeds with anti-FLAG antibody. Proteins were extracted as described in the diagram in (B). pGluA2, GluA2 precursors; βGluA2, GluA2 basic subunits. (D) The NR-P fractions were resuspended in different concentrations of DTT buffer (0–10 mM) and the supernatants were subjected to immunoblot with anti-FLAG antibody. The asterisk indicates the larger apparent molecular mass of the mGluA2-3FLAG protein. (E) Proteins extracted by 1% lactic acid after pre-extraction with different solvents and immunoblot analyses of GluA2 and mGluA2 in the transgenic seeds with anti-FLAG antibody.

Proglutelins are assembled as trimers in the ER lumen, similar to leguminous 11S globulins (Wakasa *et al.*, 2009). To investigate the assembly of mGluA2 proglutelin accumulated in the ER in the EM1317 mutant, the proteins were extracted under varying reducing conditions as described previously (Onda *et al.*, 2009). As shown in Fig. 7A, GluA2 proglutelin could be detected in the fraction extracted with globulin extraction buffer, as reported previously (Wakasa *et al.*, 2009). However, no mGluA2 could be extracted with the saline solution, suggesting that the solubility of mGluA2 had been changed. Furthermore, in the wild-type the proglutelins could be efficiently dissolved into the supernatant under non-reducing conditions (Fig. 7B, lane S). However, the extraction of proglutelins from EM1317 and *esp2* seeds required a reducing agent (Fig. 7B, lane P). To examine whether the changes in solubility of mGluA2 were due to the lack of the motif, we extracted the protein from the transgenic seeds. Immunoblot analysis indicated that the proglutelin of mGluA2-3FLAG could only be extracted under reducing conditions, while that of GluA2-3FLAG could be dissolved without a reducing agent (Fig. 7C). Similarly, the extraction of mGluB4-3FLAG proglutelin from the transgenic seeds required a reducing agent (Supplementary Fig. S7A).

To investigate whether the mGluA2 formed non-native intermolecular disulfide bonds in the non-reduced pellet (NR-P) fractions, the proteins were extracted from the pellet with different DTT concentrations and analysed by immunoblotting. As shown in Fig. 7D, the 60-kDa mGluA2-3FLAG and an apparently larger protein of molecular mass ~130 kDa were extracted by DTT in a concentration-dependent manner, whereas they were not detectable in GluA2-3FLAG fractions. Similar results were observed for mGluB4-3FLAG transgenic seeds (Supplementary Fig. S7B). We then performed SDG centrifugation to investigate the assembly of mGluA2. As shown in Supplementary Fig. S7D, GluA2 was enriched in fractions 13–15, which have been reported to be assembled into trimers (Wakasa *et al.*, 2009), while mGluA2 was detected in fractions 16–18, which may be aggregated as dimers. These results indicated that the proglutelins of mGluA2 or mGluB4 misassembled and formed aggregates in the mutant and transgenic seeds.

To detect whether mGluA2 aggregated with prolamins in the ER, we examined the conditions for proglutelin extraction as described in the *esp2* mutant (Takemoto *et al.*, 2002). As shown in Fig. 7E, GluA2 was extracted as long as the globulins were removed. However, the extraction of mGluA2 required 2-ME reducing conditions (Fig. 7E, lanes 3 and 6). Prior removal of the prolamins had no effect on proglutelin extraction (Fig. 7E, lane 5). Furthermore, prolamins with larger molecular mass (proglutelin-bonded prolamins) could not be detected in the NR-P fractions under reducing conditions (Supplementary Fig. S7C). The results of SDG centrifugation showed that the prolamins were enriched in fractions 20–22 in both the GluA2-3FLAG and mGluA2-3FLAG transgenic seeds (Supplementary Fig. S7D). These results suggested that mGluA2 formed non-native intermolecular disulfide bonds with itself, but not with prolamins.

### Disruption of intramolecular disulfide bonds does not affect the assembly of proglutelin in the ER lumen

**Fig. 8.**
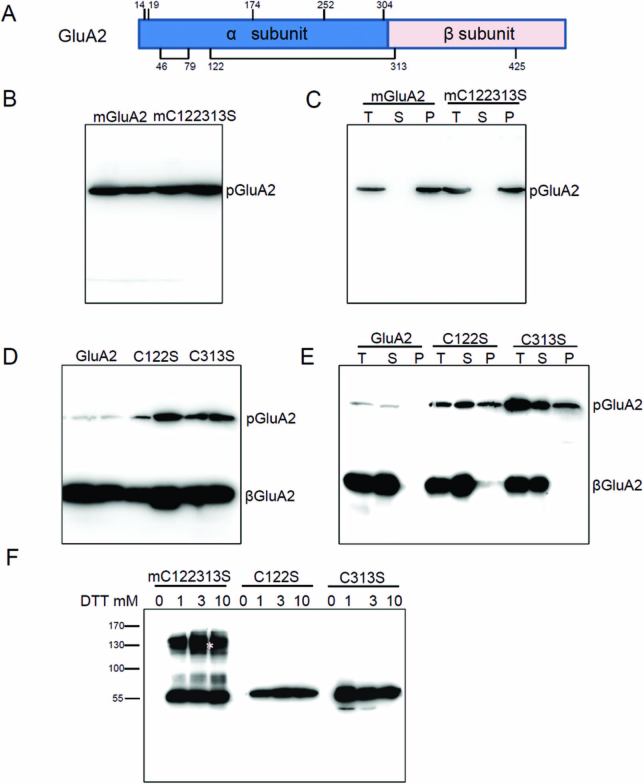
Analysis of Cys residues in GluA2. (A) Diagrammatic representation of the proposed positions of free Cys residues and disulfide bonds in GluA2. (B) Immunoblotting detects fusion protein expression with anti-FLAG antibody in mC122313S transgenic lines. pGluA2, GluA2 precursors. (C) Immunoblotting detects fusion protein expression in the T, S, and P fractions (see Fig. 7B) with anti-FLAG antibody in mC122313S transgenic lines. (D) Immunoblotting detects fusion protein expression with anti-FLAG antibody in the C122S and C313S transgenic lines. βGluA2, GluA2 basic subunits. (E) Immunoblotting detects fusion protein expression in the T, S, and P fractions with anti-FLAG antibody in the C122S and C313S transgenic lines. (F) The NR-P fractions (see Fig. 7D) were resuspended in different concentrations of DTT buffer (0–10 mM) and the supernatants were subjected to immunoblotting with anti-FLAG antibody. The asterisk indicates the larger apparent molecular mass of the mC122313S protein.

Proglutelins have been reported to form intramolecular disulfide bonds in the ER (Yamagata *et al.*, 1982). The deletion of ASLVYIIQG in GluA2 and VYIIQG in GluB4 led to aggregation of proglutelins through non-native intermolecular disulfide bonds, suggesting that this conserved motif may be essential for the correct formation of disulfide bonds. GluA2 has 10 Cys residues, the first two of which are in the signal peptide. Of the other eight, four conserved Cys residues (C_46_, C_79_, C_122_, and C_313_) are predicted to form two intramolecular disulfide bonds: Cys-122 and Cys-313 form a disulfide bond between the acidic and basic polypeptide, and Cys-46 and Cys-79 form a disulfide bond within the acidic polypeptide (Fig. 8A) (Katsube *et al.*, 1999; Katsube-Tanaka *et al.*, 2004). The highly conserved motif _105_LVYIIQGRG_113_ is close to the Cys-122 residue. It is possible that deletion of any of the nine conserved residues could lead to alterations in the crystal structure and formation of non-native intermolecular disulfide bonds instead of the correct intramolecular disulfide bonds between Cys-122 and Cys-313. To test this hypothesis, we used site-directed mutagenesis to replace Cys-122 or/and Cys-313 in GluA2-3FLAG and mGluA2-3FLAG with Ser and generated transgenic rice. The immunoblot results showed that the expressed mGluA2 with two simultaneous cysteine mutations (mC122313S) was identified as 60 kDa proglutelin (Fig. 8B). The extraction of the mC122313S protein also required a reducing agent, similar to mGluA2 (Fig. 8C). These results implied that Cys-122 and Cys-313 were not involved in the formation of non-native intermolecular disulfide bonds in mGluA2.

We also detected the fusion protein of GluA2 with a single cysteine mutation (C122S, C313S) in transgenic rice. Both exhibited the β subunit and proglutelin fused with 3FLAG bands, indicating that the C122S and C313S proteins could be transported into the PSVs and proteolytically processed into α and β mature subunits. However, the proglutelins of C122S and C313S accumulated more than that of GluA2 (Fig. 8D). Compared to GluA2, part of the C122S and C313S proglutelins were deposited in the NR-P fractions and dissolved under reducing conditions (Fig. 8E, lane P), suggesting that the intramolecular disulfide bond between Cys-122 and Cys-313 was required for correct folding of GluA2. The mC122313S formed aggregates as dimers similar to mGluA2; however, the C122S and C313S proglutelins were maintained as monomers in the NR-P fractions (Fig. 8F). These results indicated that the disruption of these intramolecular disulfide bonds was not responsible for the misassembly of mGluA2 proglutelins.

### Misassembled proglutelin induces ER stress and is degraded through the proteasome-mediated ER-associated degradation pathway

High-level accumulation of misfolded proteins in the ER induces ER stress, leading to up-regulation of molecular chaperones to maintain ER homeostasis. Persistent misfolded and misassembled secretory proteins are degraded by the ER-associated degradation (ERAD) pathway to alleviate ER stress (Liu and Howell, 2016). BiP1 and PDIL1-1 are the major chaperones responsible for correct folding of proglutelins in the lumen of the ER (Satoh-Cruz *et al.*, 2010; Wakasa *et al.*, 2011). BiP also acts as a sensor of ER stress, recognizing the degree of accumulation of misfolded or unfolded proteins in the ER (Wakasa *et al.*, 2012). The level of BiP remains consistently high in the embryos of the Arabidopsis *mag2* mutant, which abnormally accumulates the precursors of 2S albumin and 12S globulin in the ER (Li *et al.*, 2006). In the EM1317 mutant line, the accumulation of molecular chaperones was detected, and the levels of BiP1, BiP4&5, PDI1-1, PDI2-3 and CNX were found to be elevated (Supplementary Fig. S8A). In particular, the ER stress marker BiP4&5 was markedly up-regulated. These results indicated that EM1317 was under ER stress caused by the accumulation of misassembled proglutelins in the ER, which activated the unfolded-protein response (UPR) signal pathway to increase the expression of ER chaperones to facilitate protein assembly. On the other hand, immunolocalization analyses showed that mGluA2, as well as mGluA1 and mGluB4, remained and accumulated in the ER. These observations raised questions regarding whether the mutated proglutelins could be degraded. Indeed, we found that when mGluA2 was expressed in large quantities, a small fraction was degraded (Supplementary Fig. S8B). The degraded bands were only detected in proteins of mGluA2-3FLAG transgenic seeds and not in GluA2-3FLAG. To examine the pattern of degradation, we treated 3-week-old developing seeds of mGluA2-3FLAG with 20 μM MG132, a proteasome inhibitor that can prevent degradation of many ERAD substrates (Hong *et al.*, 2008). The results indicated that MG132 treatment could suppress the degradation of mGluA2 (Supplementary Fig. S8C). This suggested that misassembled proglutelins were degraded through a proteasome-mediated ERAD pathway. This was not in conflict with the observation that the mutated proglutelins accumulated in the ER lumen, as the amount of the abnormal protein probably exceeded the capacity of ERAD.

## Discussion

### Mixed accumulation of proglutelins and prolamins in the ER lumen deforms the structure of PBs

Glutelin processing and transport mutants of rice usually exhibit a common phenotype of 57-kDa proglutelin accumulation. However, the PB types at each step are distinct and can serve as indicators reflecting the step in glutelin folding, transport, and processing affected by the mutation. The PDI1-1 mutation (*esp2/pdi1-1*) blocks the proglutelins in the ER, resulting in small ER-derived protein bodies without normal PB-Is (Takemoto *et al.*, 2002). Mutations affecting the glutelin ER export step, including *glup2/gpa4/got1b* (Fukuda *et al.*, 2016; Wang *et al.*, 2016), and *Sar1*-RNAi lines (Tian *et al.*, 2013) result in ER-derived protein bodies with an electron-dense core surrounded by large amounts of protein aggregates with low electron density. In mutants with defects in transport from the Golgi apparatus to PSVs, such as *gpa1/glup4/rab5a* (Wang *et al.*, 2010; Fukuda *et al.*, 2011), *glup6/gpa2/vps9a* (Fukuda *et al.*, 2013; Liu *et al.*, 2013), and *gpa3* (Ren *et al.*, 2014), glutelins are secreted extracellularly, forming paramural bodies and reducing the size of PB-IIs but with normal PB-Is. In the *W379/glup3/vpe1* mutant, proglutelins that fail to be proteolytically processed in the PSVs form round-shaped PB-IIs that lack the typical crystalline lattice structure (Wang *et al.*, 2009; Kumamaru *et al.*, 2010). In the EM1317 mutant endosperm, normal PB-Is were not observed, and a new type of PB was formed instead. These nPB-Is were spherical and much smaller than normal PB-Is, and did not have the concentric ring structure (Fig. 1D). Furthermore, the nPB-Is contained both glutelins and prolamins (Fig. 2). These characteristics of nPB-Is in the EM1317 endosperm were similar to those in the *esp2* mutant, suggesting that the mutated GluA2 was blocked in the ER due to a defect in folding or assembly of the protein.

It has been reported that 10-kDa and 13-kDa prolamins accumulate in the central core and peripheral layers of PB-Is, respectively, thus forming the concentric ring structure of PB-Is (Nagamine *et al.*, 2011). In addition, the segregation of prolamins and proglutelins in the ER is critical for proper formation of PB-Is. In *esp2* seeds, proglutelins form complexes through intermolecular disulfide bonds with prolamins and accumulate in the ER, resulting in deformation of the structure of PB-Is (Takemoto *et al.*, 2002; Onda *et al.*, 2009). However, the mutated GluA2 formed aggregates homologously through non-native intermolecular disulfide bonds, rather than with prolamines, in the ER lumen, which also deformed the structure of the PB-Is. Similar results were observed in mGluA2 and mGluB4 transgenic seeds (Fig. 7; Supplementary Fig. S7). The accumulation of mutated proglutelins in the ER also disrupted the concentric ring structure of the PB-Is in the transgenic seeds (Figs 4–6). However, in *Sar1*-RNAi seeds, proglutelin does not exit from the ER and it forms novel PBs, while the PB-Is are mostly normal and distinct from them (Tian *et al.*, 2013). These results suggest that the correct assembly of proglutelins is critical for segregation of prolamins from proglutelins in the ER lumen, which is essential for the proper formation of PB-Is. Failing to acquire the correct conformation, proglutelins tend to be incorporated into prolamin PBs, disturbing the structure of PB-Is.

### The LVYIIQGRG motif is essential for the correct assembly of proglutelins

Previously, we reported that proglutelins are exported from the ER through the coat-protein complex-II (COPII; Tian *et al.*, 2013). our observation here that the mutated proglutelins were blocked in the ER gives rise to the question of whether the LVYIIQGRG motif acts as an ER export signal for proglutelins. To answer this question, we constructed a three-dimensional structural model of GluA2 based on the known structure of the pumpkin pro-11S globulin, which shares 42% amino acid similarity with GluA2 (Tandang-Silvas *et al.*, 2010). Homology model structure analysis predicted that the LVYIIQ sequence forms a β-sheet buried inside the structure and that the LVYIIQGRG motif is not exposed at the protein surface (Supplementary Fig. S9). The LVYIIQGRG motif does not contain the classical di-acidic ([D/E]×[E/D]) or di-hydrophobic (FF, FY) ER export signals identified in plant transmembrane proteins. Moreover, the phenotype of nPB-Is in the EM1317 mutant was different from that in *got1b* and *Sar1*-RNAi lines that are defective in COPII, but it was similar to that in the *esp2* mutant, suggesting that the mutated GluA2 was defective in protein folding or assembly. The LVYIIQGRG motif was fused to the N-terminus of GFP under the control of the *35S* promoter and a signal peptide of GluA2. The construct was transiently expressed in rice protoplasts, and we found that the LVYIIQGRG motif could not guide GFP to exit from the ER (Supplementary Fig. S10). Therefore, this motif may not act as an ER export signal recognized by Sec24.

Not all amino acid sequence changes in glutelin affect its transport. The C-terminus of the glutelin acidic subunit is called the hypervariable region (HVR), where the length and amino acid composition vary markedly among the 11S globulins. It has been reported that insertion of 150 residues into HVR does not affect its transport to PB-IIs and that it is processed normally (Yasuda *et al.*, 2006). Expression of unprocessed proglutelin with a mutation in the conserved processing site (Asn-Gly) at the junction between the acidic and basic chains does not affect protein trafficking and accumulation (Wakasa *et al.*, 2009). In our study, LVYIIQGRG-deleted GluA2 was blocked in the ER. Amino acid sequence alignment of GluA2 and other 11S globulin family members revealed that some residues of the LVYIIQGRG motif were conserved in the 11S globulin family (Supplementary Fig. S11). Crystal structure analysis of soybean proA3B4 indicated that Gly-74 is involved in correct globular folding (Tandang-Silvas *et al.*, 2010). The corresponding glycine in GluA2 is Gly-111, which is located in the conserved motif. This suggests that Gly-111 and the surrounding residues may play important roles in maintaining correct folding and in assembly of proglutelins.

Proglutelins are assembled as trimers in the ER lumen, similar to leguminous 11S globulins (Wakasa *et al.*, 2009). However, most mGluA2 or mGluB4 aggregated as dimers through non-native intermolecular disulfide bonds. The solubility of the mutated proglutelins also changed and the mGluA2 proglutelin could not be extracted with saline solution, and its dissolution depended on the reducing agent (Fig. 7). These results indicated that the deleted amino acids were necessary for correct assembly of proglutelins. Internal regions that are necessary for protein assembly have been reported in phaseolin (Foresti *et al.*, 2003); however, no sequence motif has been reported in rice glutelins. The present study extends our understanding of protein folding, assembly, and export in plants.

### Intramolecular disulfide bonds are not necessary for proglutelin assembly

It has been reported that malformation of intermolecular disulfide bonds between globulin and prolamins in the ER lumen results in the retention of globulin in ER PBs (Kawagoe *et al.*, 2005). In *esp2/pdi1-1* proglutelins are assumed to co-assemble with prolamins via intermolecular disulfide bonds to form intracisternal aggregates within the ER (Takemoto *et al.*, 2002). In contrast with *esp2*, the expression level of PDI1-1 was increased in the EM1317 mutant endosperm (Supplementary Fig. S8). Although a high-mass proglutelin was detected in the NR-P fractions, proteins with the mass of prolamin (proglutelin-bonded prolamin) could not be detected, and the prolamins and mGluA2 were enriched in different fractions following SDG centrifugation (Supplementary Fig. S7D). These results indicated that the non-native intermolecular disulfide bonds were formed among mutated glutelin molecules rather than between mutated glutelin and prolamin molecules, which explains why only the mutated proglutelins were blocked in the ER. This is not surprising because glutelin and prolamin are synthesized in the cisternal and PB-ER, respectively (Washida *et al.*, 2012), and PDIL1-1 is predominantly located in the cisternal ER rather than distributed on PB-ER (Satoh-Cruz *et al.*, 2010). The nascent proglutelin is initially bound with PDI and therefore it is less likely to bind with prolamin. Furthermore, it has been reported that folding drives the formation of disulfide bonds, and the pairing of cysteines is determined by substrate protein rather than by PDI (Wilkinson and Gilbert, 2004; Kosuri *et al.*, 2012; Qin *et al.*, 2015).

Trimerization of proglutelins in the ER lumen is essential for their transport into the PSVs. In the process, it has been proposed that GluA proglutelins undergo heteroassembly with GluB (Wakasa *et al.*, 2009). If the mutated proglutelins assemble with other proglutelins, it may result in transport of the mutant protein to the PSVs due to the presence of export signals in the wild-type proglutelins. Otherwise, the normal proglutelins would also be blocked in the ER due to improper conformation with the mutated proglutelin. However, only the proglutelins with the mutated motif (mGluA2, mGluA1, or mGluB4) were blocked in the ER, while the wild-type proglutelins were sorted to the PSVs (Fig. 2, 4, 5). These results suggested that proglutelin was assembled homologously as a trimer in the ER lumen. The simultaneous mutation of Cys-122 and Cys-313 in mGluA2 did not recover the ER export or PSV-targeting of the protein, and the mutated proglutelins remained aggregated through intermolecular disulfide bonds (Fig. 8F). On the other hand, disruption of the intramolecular disulfide bond by a single cysteine mutation (C122S or C313S) in GluA2 did not cause a larger molecular mass aggregate of the proglutelins (Fig. 8F). These results suggested that the intramolecular disulfide bonds may not be essential for the correct assembly of proglutelin to trimers, and the trimerization of proglutelins may be mediated through hydrophobic interaction. This is supported by reports that disrupted inter- and intra-chain disulfide bonds of glycinin and legumin do not prevent their assembly into trimers (Utsumi *et al.*, 1993; Jung *et al.*, 1998). Deletion of the motif may disrupt the hydrophobic interaction leading to the misassembly of proglutelins. It is notable that GluA2 with a single cysteine mutation (C122S or C313S) accumulated more proglutelins. Although the extraction of these proglutelins required reducing conditions, they were maintained as monomers. These results indicated that the intramolecular disulfide bond between Cys-122 and Cys-313 is important for the correct folding of proglutelins, which may in turn affect their transport.

## Supplementary data

Supplementary data are available at *JXB* online.

Fig. S1. Morphology and analyses of storage proteins in the EM1317 mutant line.

Fig. S2. Diagram of deleted amino acid constructs in rice protoplasts.

Fig. S3. Subcellular localization of GluA2 and mGluA2 in rice protoplasts.

Fig. S4. Immunolocalization of the GluA1-/GluB4-3FLAG fusion proteins in transgenic endosperm.

Fig. S5. Subcellular localization of ΔASL and ΔVYIIQG in rice protoplasts.

Fig. S6. Subcellular localization of GluA2 with deletion of one amino acid in rice protoplasts.

Fig. S7. Aggregation of the mGluB4 form through non-native intermolecular disulfide bonds.

Fig. S8. Degradation of a few mGluA2 proteins by a proteasome-mediated ER-associated degradation pathway.

Fig. S9. Three-dimensional model of GluA2.

Fig. S10. Subcellular localization of LVYIIQGRG-GFP.

Fig. S11. Amino acid sequence alignment of GluA2 and other 11S globulin family members.

Table S1. Primer sequences used in this study.

Table S2. Concentrations of amino acids in finely ground seeds.

Supplementary Material
