# Supplementary material for: A conserved motif is essential for the correct assembly of proglutelins and for their export from the endoplasmic reticulum in rice endosperm
Source: J Exp Bot. 2018 Aug 10;69(21):5029–43. doi: 10.1093/jxb/ery290 (PMC6184509; doi:10.1093/jxb/ery290)
Supplement: Supplementary Material [file ery290_suppl_supplementary_materials.pdf]

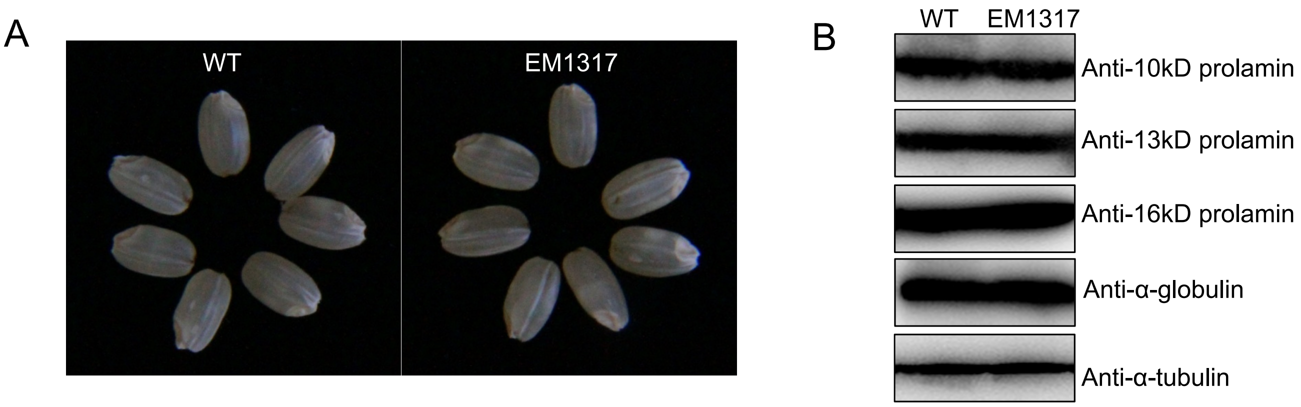

Supplemental Figure S1. Morphology and storage proteins analyses of the EM1317 mutant.
(A) Morphology of mature seeds from WT and the EM1317 mutant.
(B) Immunoblot analysis of the prolamins (10kD, 13kD, 16kD) and α-globulin proteins. The α-tubulin was used as a loading control.

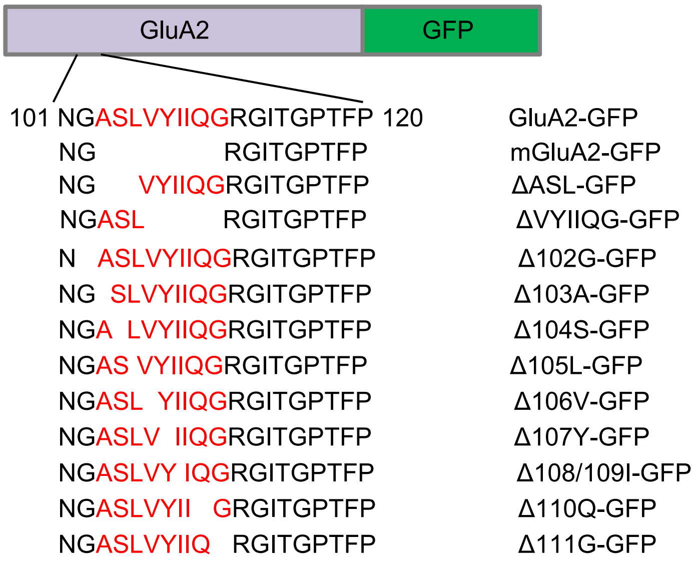

Supplemental Figure S2. Scheme of deleted amino acid constructs in rice protoplasts.

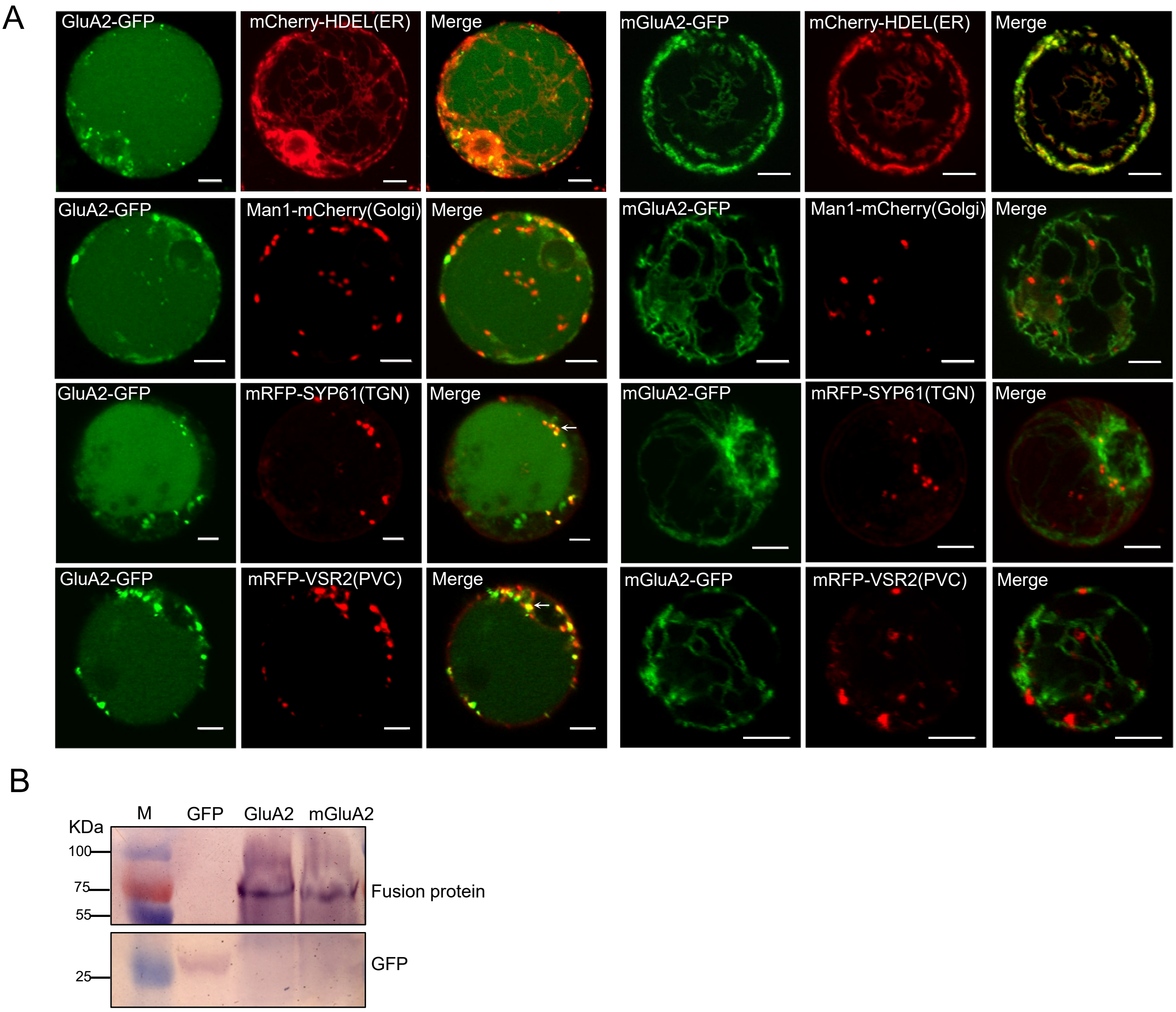

Supplemental Figure S3. Subcellular localization of GluA2 and mGluA2 in rice protoplasts.

(A) Rice protoplasts cell were cotransformed with GluA2, mGluA2 and ER marker mCherry–HDEL, Golgi marker GmMan1-mCherry, TGN marker mRFP-SYP61, PVC marker mRFP-VSR2, respectively. The panel shows the confocal images of the GFP signal (green), the mCherry signal (red), and the merged GFP-mCherry signal. The white arrows indicate the punctate structures of GluA2-GFP co-localized with the TGN and PVC marker. Scale bars = 5 μm.

(B) Immunoblot detected the fusion protein with anti-GFP antibody in GluA2-GFP and mGluA2-GFP transformed rice protoplasts cell. The GFP under the control of the CaMV 35S promoter was the control.

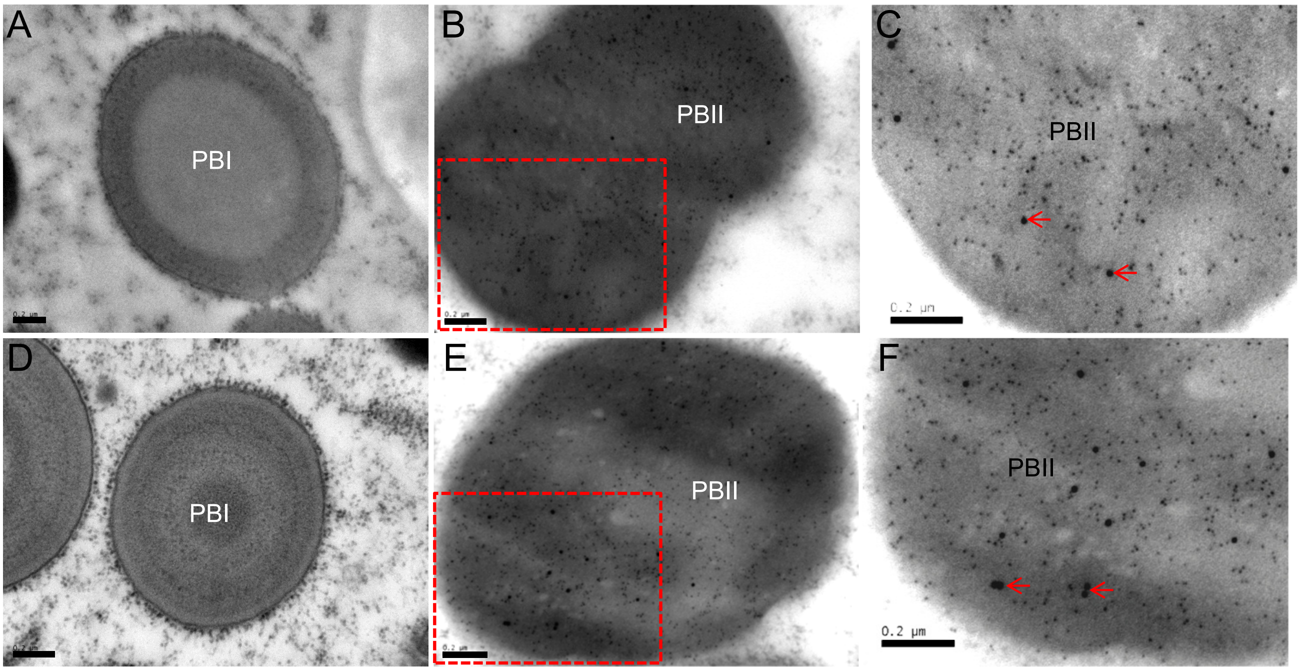

Supplemental Figure S4. Immunolocalization of the GluA1/GluB4-3FLAG fusion protein in transgenic endosperm.

The glutelin and FLAG antibodies were labelled with 5-nm and 15-nm immunogold particles, respectively, in GluA1-3FLAG (A-C) and GluB4-3FLAG (D-F) transgenic endosperm. (C, F) are enlarged images of areas inside the red boxes in (B, E). The red arrows indicated the 15-nm immunogold particles labelled fusion protein were in PB-II. Scale bars = 0.2 μm.

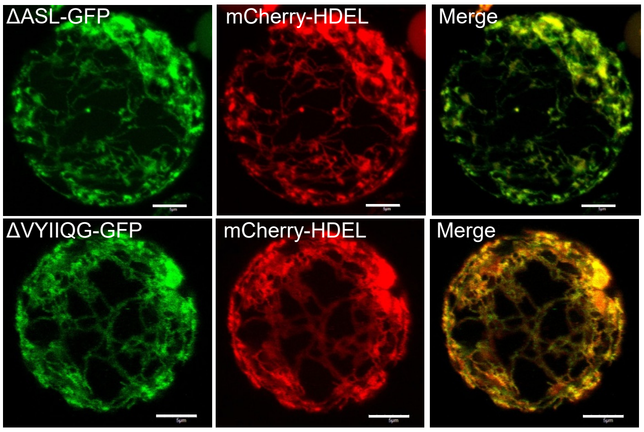

Supplemental Figure S5. Subcellular localization of ΔASL and ΔVYIIQG in rice protoplasts.
Rice protoplasts cell were cotransformed with ΔASL, ΔVYIIQG and ER marker mCherry–HDEL, respectively. Scale bars = 5 μm.

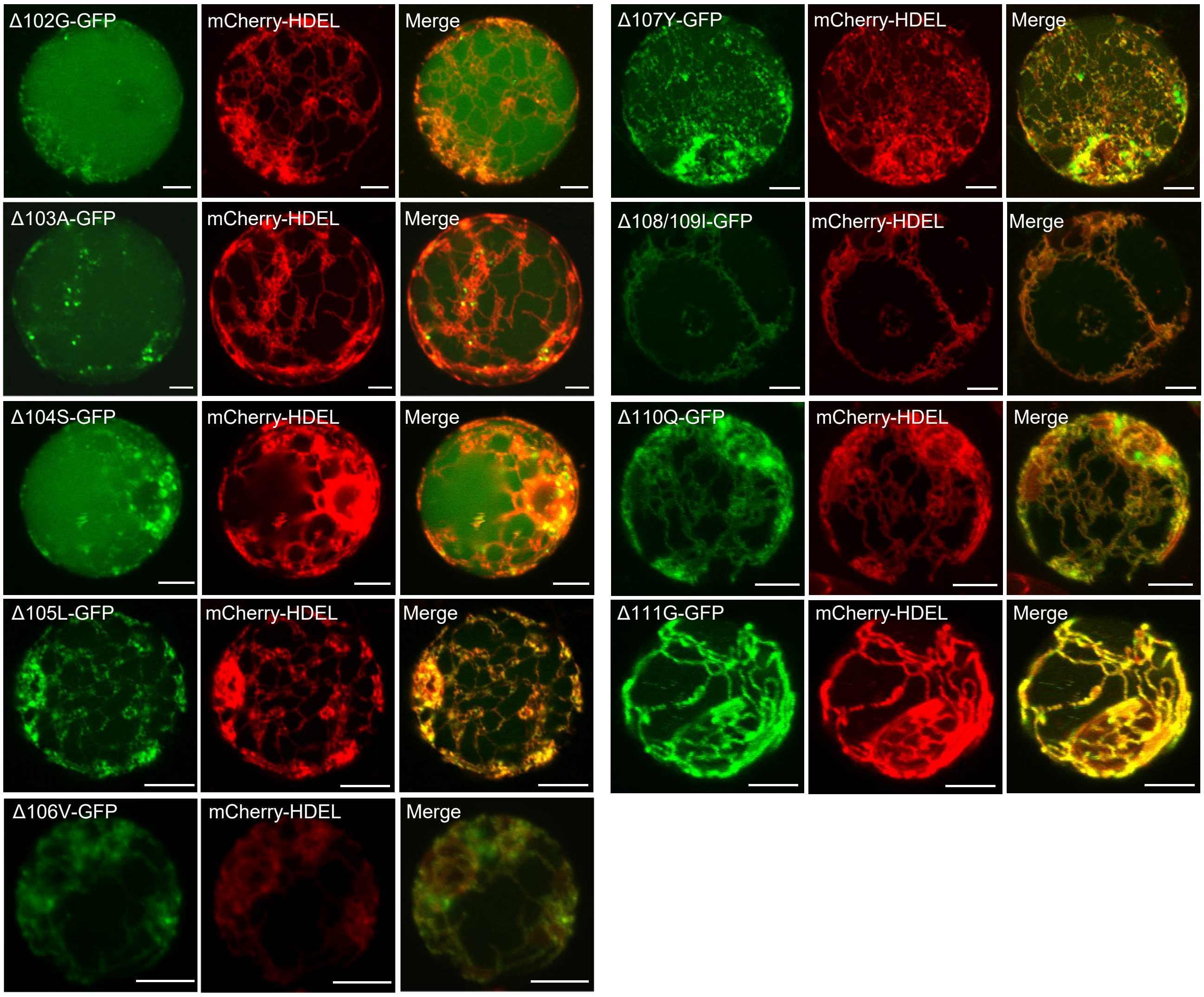

Supplemental Figure S6. Subcellular localization of GluA2 with deletion of one amino acid in rice protoplasts. Deletion of 102G, 103A and 104S, GluA2 export from ER, but deletion of 105L,106V, 107Y, 108/109I, 110Q and 111G, GluA2 retention in the ER. Scale bars = 5 µm.

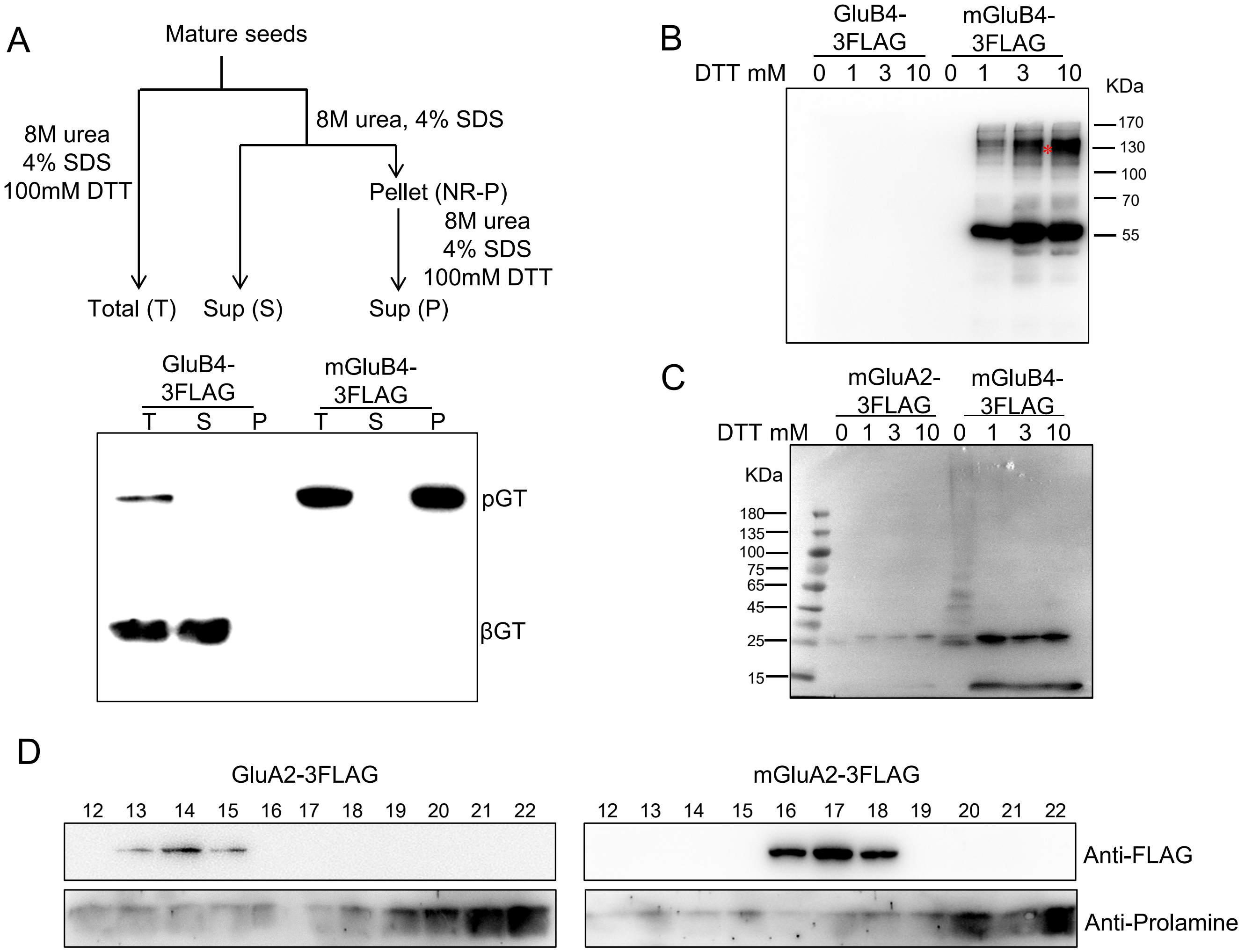

Supplemental Figure S7. The mGluB4 form aggregated through non-native intermolecular disulfide bonds. Proteins were extracted from the mature seeds as. The T, S, and P fractions were subjected to SDS-PAGE, followed by CBB staining or immunoblot. pGT, glutelin precursors; βGT, glutelin basic subunits.

(A) Immunoblot detected the protein of GluB4/mGluB4-3FLAG transgenic seeds with anti-FLAG antibody. Proteins were extracted as described in schematic representation.

(B) NR-P fractions were resuspended as described in Figure 8. The supernatants were performed to immunoblot with anti-FLAG antibody. The asterisks indicate the larger apparent molecular mass of mGluB4-3FLAG protein.

(C) Immunoblot detected whether the prolamin assembled with mutated glutelin in the NR-P fractions with anti-prolamin antibody. The result indicated only monomer and dimer bands of prolamin, but not prolamin bands with larger molecular mass. This suggested the formation of nonnative intermolecular disulfide bonds were not between glutelin and prolamin.

(D) Assembly of GluA2 and mGluA2 in transgenic seeds. The fractions of SDG centrifugation were analysed by immunoblot using anti-FLAG and anti-prolamin antibodies.

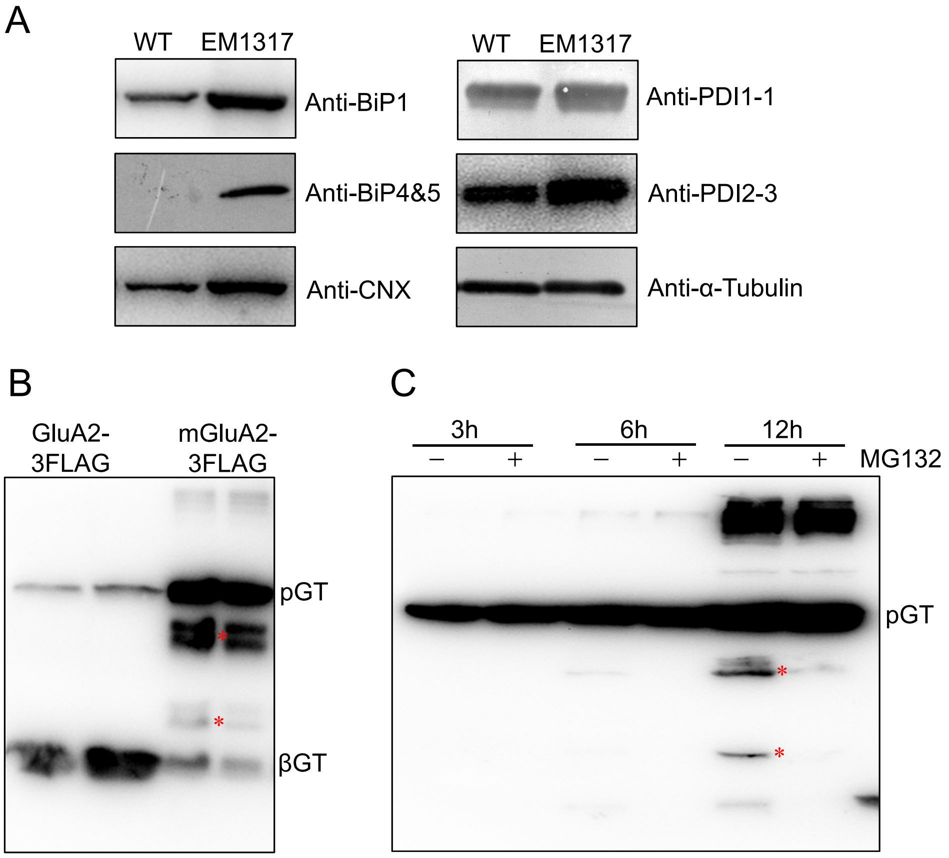

Supplemental Figure S8. A few mGluA2 proteins were degraded by a proteasome-mediated ER-associated degradation (ERAD) pathway. Asterisks indicate degradation products of mGluA2 proteins.

(A) Immunoblot analysis of ER chaperones in the wild type and EM1317 mature seeds. The α-tubulin was used as a loading control.

(B) Immunoblot detected the proteins of GluA2/mGluA2-3FLAG transgenic seeds with anti-FLAG antibody. pGT, glutelin precursors; βGT, glutelin basic subunits.

(C) Immunoblot analysis of mGluA2 proteins in 3-week-old developing seeds of an mGluA2-3FLAG transgenic line treated with or without 20 μM MG132.

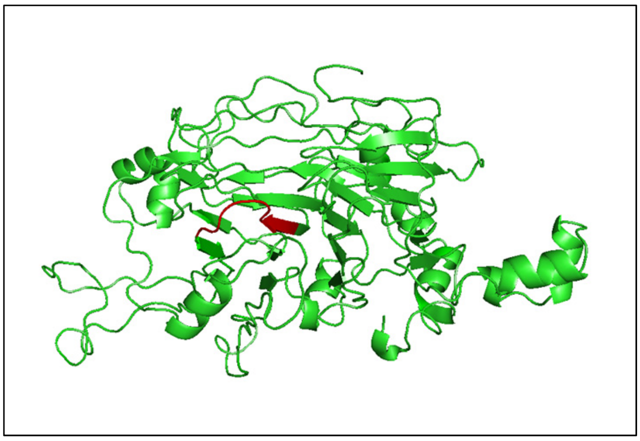

Supplemental Figure S9. Three-dimensional model of GluA2. The model was constructed on the basis of amino acid similarity with the pro-11S globulin of pumpkin [Protein Data Bank (PDB) 2E9Q]. The LVYIIQGRG motif was highlighted in red, LVYIIQ among them was predicted to be a β-sheet.

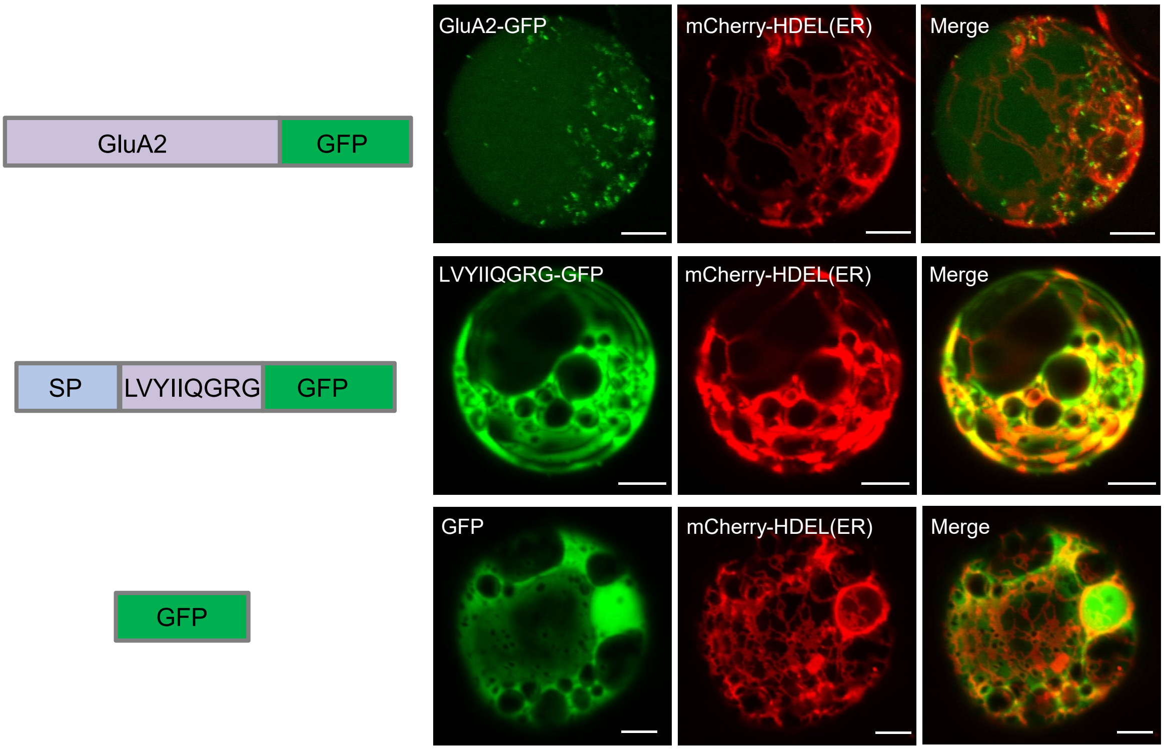

Supplemental Figure S10. Subcellular localization of LVYIIQGRG-GFP.
Rice protoplasts cell were cotransformed with GluA2-GFP, LVYIIQGRG-GFP and GFP control and ER marker mCherry–HDEL, respectively. The LVYIIQGRG-GFP fusion protein was translocated into the ER since it contains the GluA2 signal peptide (SP). If the LVYIIQGRG is an ER export signal, the fusion protein could exit from the ER. However, the green fluorescence of LVYIIQGRG-GFP merged with the red fluorescence of the mCherry-HDEL, showing that fusion protein was blocked in the ER. This result suggested that the LVYIIQGRG motif may not act as an ER export signal. Scale bars = 5 μm.

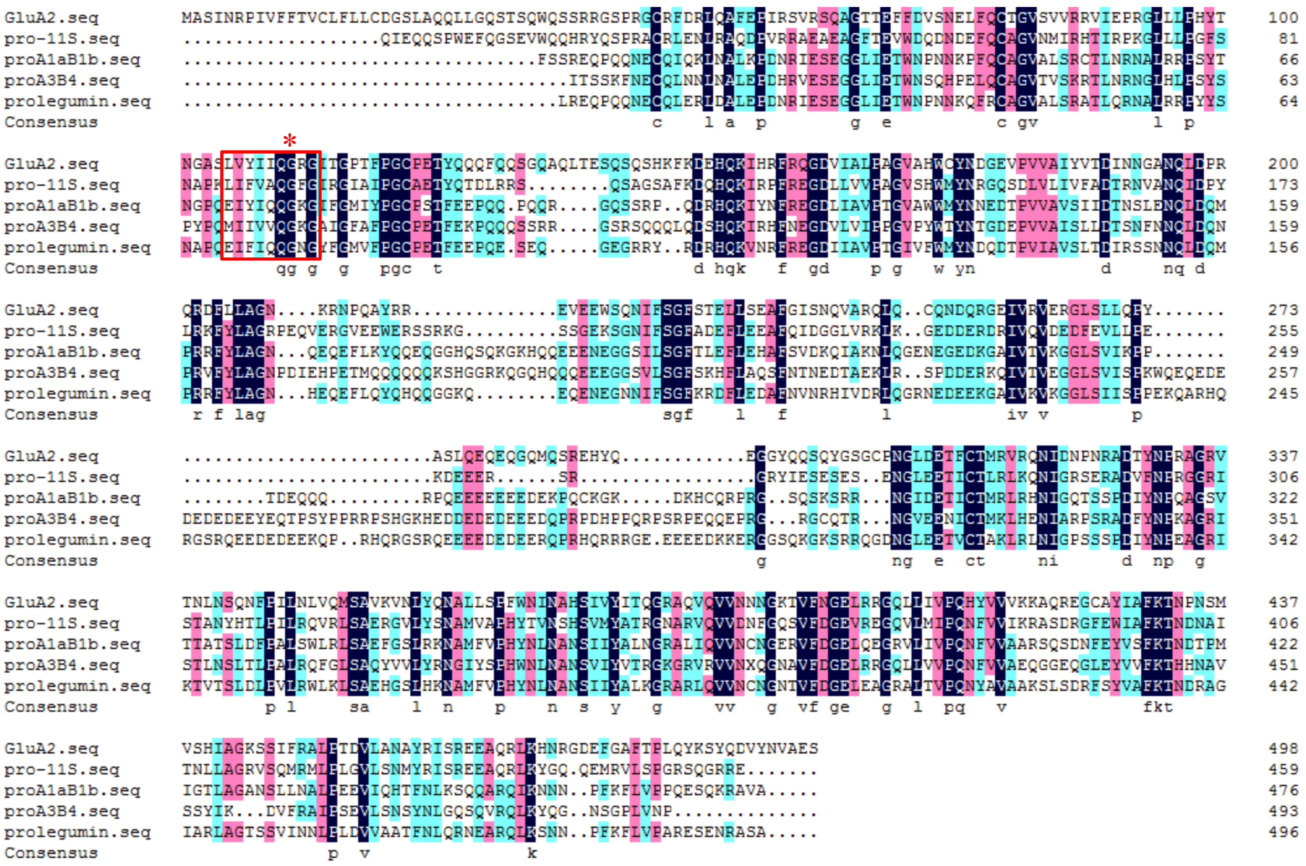

Supplemental Figure S11. Amino acid sequence alignment of GluA2 and other 11S globulin family members: pro-11S in pumpkin, prolegumin in pea, proA1aB1b and proA3B4 in soybean. Boxed letters indicate the identified nine amino acids. * labelled Gly74 in proA3B4.

Supplemental Table S1 Primer sequences used in this study

Experiment	Primer name	Sequence
Gene Cloning	GluA1-F	5'- CAAGTTCATTAGTACTACAACAACATGGC-3'
	GluA1-R	5'- AAGGATAAAGAGAGGCTTTATCATCA-3'
	GluA2-F	5'-ATGGCATCCATAAATCGCCCC-3'
	GluA2-R	5'-AGAGGATTCCGCCACATTATAAACG-3'
	GluA3-F	5'-TCACAAAAGCATTGAGTTCAGT-3'
	GluA3-R	5'-TTATGCACTCACAGATATGTCTTGG-3'
	GluA2-Pro-F	5'- ATTACTATCTGAGCATTCCCC-3'
	GluA2-Pro-R	5'-GTTGTTGTAGGACTAATGAAC-3'
	GluA2-3UTR-F	5'-GTTGGCAATGCGGATAAAGA-3'
	GluA2-3UTR-R	5'-GGTTGTCCATTCTCTGTTTTCTCAT-3'
Binary vectors construction	GluA2/mGluA2 -GPTV-F	5'- ACGCGTCGACATGGCATCCATAAATCGCCC-3'
	GluA2/mGluA2 -GPTV-R	5'TTATCGTCGTCATCCTTGTAATCCCCGGGAGAGGATTC CGCCACATTATAAAC-3'
	GluA1-GPTV-F	5'- ACGCGTCGACATGGCATCCATAAATCGCC-3'
	GluA1-GPTV-R	5'- AGAGGATTCTGCCGCATTATAAA-3'
	GluB4-GPTV-F	5'- ACGCGTCGACATGGCGACCATAGCTTTCTCT-3'
	GluB4-GPTV-R	5'- TCCCCCGGGCTCTAAGGCCTCGTTCTCCG-3'
Transient expression vector construction	GluA2/mGluA2 -pBI221-F	5'- TGCTCTAGAATGGCATCCATAAATCGCCCC-3'
	GluA2/mGluA2 -pBI221-R	5'- CCCCCCGGGAGAGGATTCCGCCACATTATAAACG-3'
Deletion mutant construction	mGluA1-F	5'- AGAGGTATAACAGGGCCAACTTTC-3'
	mGluA1-R	5'- ACCATTAGTGTAATGGGGTAGTAGAA-3'
	mGluB4-F	5'- AGAGGTTCTATGGGATTAACTTTCCC-3'
	mGluB4-R	5'- CATGCCAGGAGTATTGCTGTATC-3'
	ΔAS-F	5'- CTAGTATATATCATCCAAGGGAGAGG-3'
	ΔAS-R	5'- ACCATTAGTGTAATGGGGTAGTA-3'
	ΔASL-F	5'- GTATATATCATCCAAGGGAGAG-3'
	ΔASL-R	5'- ACCATTAGTGTAATGGGGTAGTA-3'
	ΔVY-F	5'- ATCATCCAAGGGAGAGGTATAA-3'
	ΔVY-R	5'- TAGAGATGCACCATTAGTGTAAT-3'
	ΔII-F	5'- CAAGGGAGAGGTATAACAGGG-3'
	ΔII-R	5'- ATATACTAGAGATGCACCATTAGTG-3'
	ΔQG-F	5'- AGAGGTATAACAGGGCCGAC-3'
	ΔQG-R	5'- GATGATATATACTAGAGATGCACCATT-3'
	ΔRG-F	5'- ATAACAGGGCCGACTTTCCC-3'
	ΔRG-R	5'- CCCTTGGATGATATATACTAGAGATGC-3'
	ΔVYIIQG-F	5'- AGAGGTATAACAGGGCCGAC-3'
	ΔVYIIQG-R	5'- TAGAGATGCACCATTAGTGTAAT-3'
	Δ102G-F	5'- GCATCTCTAGTATATATCATCCAAGG-3'

Deletion mutant construction	Δ102G-R	5'- ATTAGTGTAATGGGGTAGTAGTAGGC-3'
	Δ103A-F	5'- TCTCTAGTATATATCATCCAAGGGAG-3'
	Δ103A-R	5'- ACCATTAGTGTAATGGGGTAGTAG-3'
	Δ104S-F	5'- CTAGTATATATCATCCAAGGGAGAGG-3'
	Δ104S-R	5'- TGCACCATTAGTGTAATGGG-3'
	Δ105L-F	5'- GTATATATCATCCAAGGGAGAGG-3'
	Δ105L-R	5'- AGATGCACCATTAGTGTAATGG-3'
	Δ106V-F	5'- TATATCATCCAAGGGAGAGGTATAAC-3'
	Δ106V-R	5'- TAGAGATGCACCATTAGTGTAATGG-3'
	Δ107Y-F	5'- ATCATCCAAGGGAGAGGTATAACAG-3'
	Δ107Y-R	5'- TACTAGAGATGCACCATTAGTGTAATGG-3'
	Δ108/109I-F	5'- ATCCAAGGGAGAGGTATAACAG-3'
	Δ108/109I-R	5'- ATATACTAGAGATGCACCATTAGTG-3'
	Δ110Q-F	5'- GGGAGAGGTATAACAGGGCC-3'
	Δ110Q-R	5'- GATGATATATACTAGAGATGCACCATT-3'
	Δ111G-F	5'- AGAGGTATAACAGGGCCGAC-3'
	Δ111G-R	5'- TTGGATGATATATACTAGAGATGCACC-3'
Substitution Cys construction	C122S-F	5'- CTCCTGAGACCTACCAGCAGCA-3'
	C122S-R	5'- AGCCTGGGAAAGTCGGCCC-3'
	C313S-F	5'- CACCATGAGGGTAAGGCAAA-3'
	C313S-R	5'- GAAAAGGTCTCATCCAAACCG-3'

Supplemental Table S2 Concentrations (mg g^{-1}) of amino acids in milled seeds

amino acids	WT	EM1317
Asp	7.26 ±0.25	7.16 ±0.11
Thr	2.75 ±0.07	2.72 ±0.01
Ser	3.44 ±0.10	3.31 ±0.04
Glu	11.04 ±0.33	10.49 ±0.27
Pro	4.67 ±0.22	4.21 ±0.05
Gly	3.63 ±0.18	3.45 ±0.03
Ala	4.54 ±0.20	4.53 ±0.09
Val	4.32 ±0.09	4.25 ±0.09
Met	1.12 ±0.05	1.13 ±0.22
Ile	3.05 ±0.15	3.13 ±0.21
Leu	6.06 ±0.25	6.08 ±0.04
Phe	3.95 ±0.18	3.94 ±0.02
His	2.41 ±0.09	2.39 ±0.13
Lys	3.48 ±0.10	3.47 ±0.02
Arg	6.63 ±0.23	5.85 ±0.25
